# Antibodies against Proinsulin and Homologous MAP Epitopes Are Detectable in Hashimoto’s Thyroiditis Sardinian Patients, an Additional Link of Association

**DOI:** 10.1371/journal.pone.0133497

**Published:** 2015-07-20

**Authors:** Magdalena Niegowska, Daniela Paccagnini, Carlo Burrai, Mario Palermo, Leonardo A. Sechi

**Affiliations:** 1 Università degli Studi di Sassari, Dipartimento di Scienze Biomediche, Sezione di Microbiologia e Virologia, Sassari; 2 Department of Endocrinology, Azienda Sanitaria Locale (ASL) 1, Sassari, Italy; Northwestern University Feinberg School of Medicine, UNITED STATES

## Abstract

Hashimoto’s thyroiditis (HT) is the prevailing organ-specific autoimmune disease in Sardinia, often complicated with other autoimmune disorders, most commonly type 1 diabetes (T1D). While numerous studies describe levels of anty-thyroid antibodies (Abs) in T1D patients, few papers evaluate the status of anti-islet autoimmunity in subjects affected by HT. Previously, we portrayed *Mycobacterium avium* subspecies *paratuberculosis* (MAP) as an environmental factor strongly associated with both diseases. In this study, we analyzed plasma of Sardinian HT patients (n=177) and healthy controls (HCs; n=175) for the presence of Abs against proinsulin and MAP-derived homologous epitopes: MAP1,4αgbp_157-173_/PI_64-80_ were recognized by 5,08% and 18,64% of HT vs 0,57% and 7,43% of HCs (AUC=0,6 for both; *p*<0,0003 and 0,002, respectively), whereas the prevalence of Abs against MAP2404c_70-85_/PI_46-61_ peptides was higher but not significant in patients when compared to HCs. In women (n=152), Abs against MAP1,4αgbp_157-173_ were detected in 12,50% of HT vs 2,75% of HCs (AUC=0,63; *p*<0,0002), while positivity to its human homolog PI_64-80_ was observed in 16,42% of HT vs 6,42% of HCs (AUC=0,61; *p*<0,001). In men (n=25), a significant anti-PI_46-61_ Abs levels were detected in 4% of HT vs none of the HCs (AUC=0,7; *p*<0,003). Age-related analyses revealed the highest prevalence between 31-40 years old (45,83%) in the total study population and among males (33,33%); in contrast, women had a higher seroreactivity between 51-60 years (42,11%). A further follow-up and determination of anti-islet Abs levels is needed to evaluate the association of immune responses directed against the MAP/PI homologous peptides with progression to overt diabetes in HT subjects.

## Introduction

Hashimoto’s thyroiditis (HT) is an autoimmune inflammation of the thyroid gland associated with alterations in glucose and insulin metabolism, therefore frequently occurring along with type 1 diabetes (T1D). Both diseases are organ-specific, characterized by T-cell infiltration, cell-mediated immunity and production of autoantibodies (aAbs) leading to dysfunction or destruction of the target organ (thyroid gland and pancreatic islet cells, respectively). It is estimated that about 20% of T1D patients is positive to anti-thyroid aAbs with prevalence in females and, inversely, 2.3% of children with autoimmune thyroiditis (AITD) have aAbs against β-cells [1–4]. However, recommended measurements of thyroid-stimulating hormone (TSH), as well as thyroid peroxidase (TPO) and thyroglobulin (Tg) aAbs levels in T1D patients to evaluate future development of overt thyroiditis is not yet a common practice [5, 6]. Similarly to other autoimmune diseases, HT and T1D have multiple etiology resulting from an interaction between genetic predisposition and environmental risk factors. Although they have been investigated for decades, there is no clear explanation concerning mechanisms underlying the development of multiple autoimmune diseases within the same individual or family [7–9].

The functioning of the thyroid gland can be impared by deficiency of trace elements such as zinc [10], essential either in follicles of the thyroid gland or for a correct maturation and secretion of insulin stored as hexameric complexes bound by two Zn^2+^ ions [11]. ZnT8 is a zinc transporter membrane protein expressed in insulin secretory granules of pancreas. It is targeted by aAbs in T1D patients and has been identified as a novel T1D biomarker increasing diagnostic sensitivity when analysed together with other T1D aAbs (ICA, IAA, IA2A and GADA) [12]. Recently, high levels of anti-ZnT8 aAbs (ZnT8A) have been associated with the onset of autoimmune thyroiditis in LADA patients [13]. Previously, we reported a significantly high positivity to ZnT8A and antibodies against homologous peptides derived from *Mycobacterium avium subsp*. *paratuberculosis* (MAP) in HT and T1D patients that corroborates a possible association of the bacterium with both diseases [14–17]. Furthermore, our group identified MAP peptides homologous to proinsulin (PI) that were highly recognized in new-onset T1D children from continental Italy (45,7–49,1%) compared to healthy controls (3,3%), hypothesizing a cross-reaction between Abs targeting the homologous PI/MAP region [18]. PI has been described as a β-cell antigen initiating T1D pathogenesis in NOD mouse [19] and Abs against its processed form, insulin, are the first to be detected in T1D patients [20].

While the scientific literature includes numerous studies describing levels of anti-thyroid Abs in T1D patients, only few papers evaluate the status of anti-islet autoimmunity in subjects affected by AITD, in particular related to specific epitopes. The above-mentioned findings encouraged us to design the current study, aimed at verifying the presence of anti-PI and homologous anti-MAP Abs in HT patients, possibly predicting a future implication of type 1 diabetes in these subjects.

## Materials and Methods

### Subjects

177 subjects (n = 25 males, n = 152 females; mean age 44,94±15,65 years) attending the Department of Endocrinology, University Hospital of Sassari (Italy), affected by HT and 175 age/sex-matched healthy controls (HCs; n = 66 males, n = 109 females; mean age 42,18±13,23 years) were enrolled in this study. HCs were blood donors at the University Hospital of Sassari with no clinical evidence of T1D or other autoimmune diseases. The patients were diagnosed based on the presence of anti-thyroid peroxidase (TPO) and anti-thyroglobulin (TG) Abs, as well as levels of thyroid-stimulating hormone (TSH), free triiodothyronine (FT3) and thyroxine (FT4). Plasma was separated by sedimentation method from venous blood samples collected in EDTA-coated BD Vacutainer tubes, and stored at -20°C.

### Peptides

MAP1,4αgbp_157-173_ (GTVELLGGPLAHPFQPL) and MAP2404c_70-85_ (RGFVVLPVTRRDVTDV) with their respective homologs PI_64-80_ (GQVELGGGPGAGSLQPL) and PI_46-61_ (RGFFYTPKTRREAEDL) were synthesized at 85,58–92,70% purity (LifeTein, South Plainfield, NJ 07080, USA) assessed by HPLC and stored in single-use aliquots at -20°C.

### ELISA

Indirect enzyme-linked immunosorbent assays (ELISA) to detect antibodies against MAP/PI homologous peptides in plasma samples were performed as described previously [18]. Highly positive samples with reactivity set at 1.000 arbitrary units (AU)/ml were used to normalize the OD values. The statistical analyses of the assay were performed using Version 6.0 Graphpad Prism software and their significance was determined through the Mann-Whitney *U* test for not normally distributed data or the Student’s *t*-test (95% CI).

### Auto-antibodies assays and thyroid hormone levels

Anti-thyroid aAbs titers were measured by chemiluminescence methodology in the serum of all HT patients, using the Liaison Anti-TPO kit (DiaSorin, Italy) for anti-TPO assay and the Liaison Anti-TG kit (DiaSorin, Italy) for anti-TG assay with normal values ranging from 0–100 UI/mL and 0–10 UI/mL, respectively, according to the manufacturer’s instructions.

Levels of FT4 (ng/dl), FT3 (pg/ml), TSH (μU/ml) were measured by the LIAISON Analyzer family (Diasorin S.p.A., Vercelli Italy).

Correlation analyses were performed with Version 6.0 Graphpad Prism software.

### Ethical statement

The current study protocol was approved by the Bioethical Committee of the University of Sassari. All participants were enrolled upon signing written informed consent. All governmental and institutional regulations regarding the ethical involvment of human volunteers in clinical studies were respected.

## Results

### Prevalence of Abs against MAP1,4αgbp and MAP2404c/PI epitopes in HT subjects and age/sex-matched HCs

**Fig 1:**
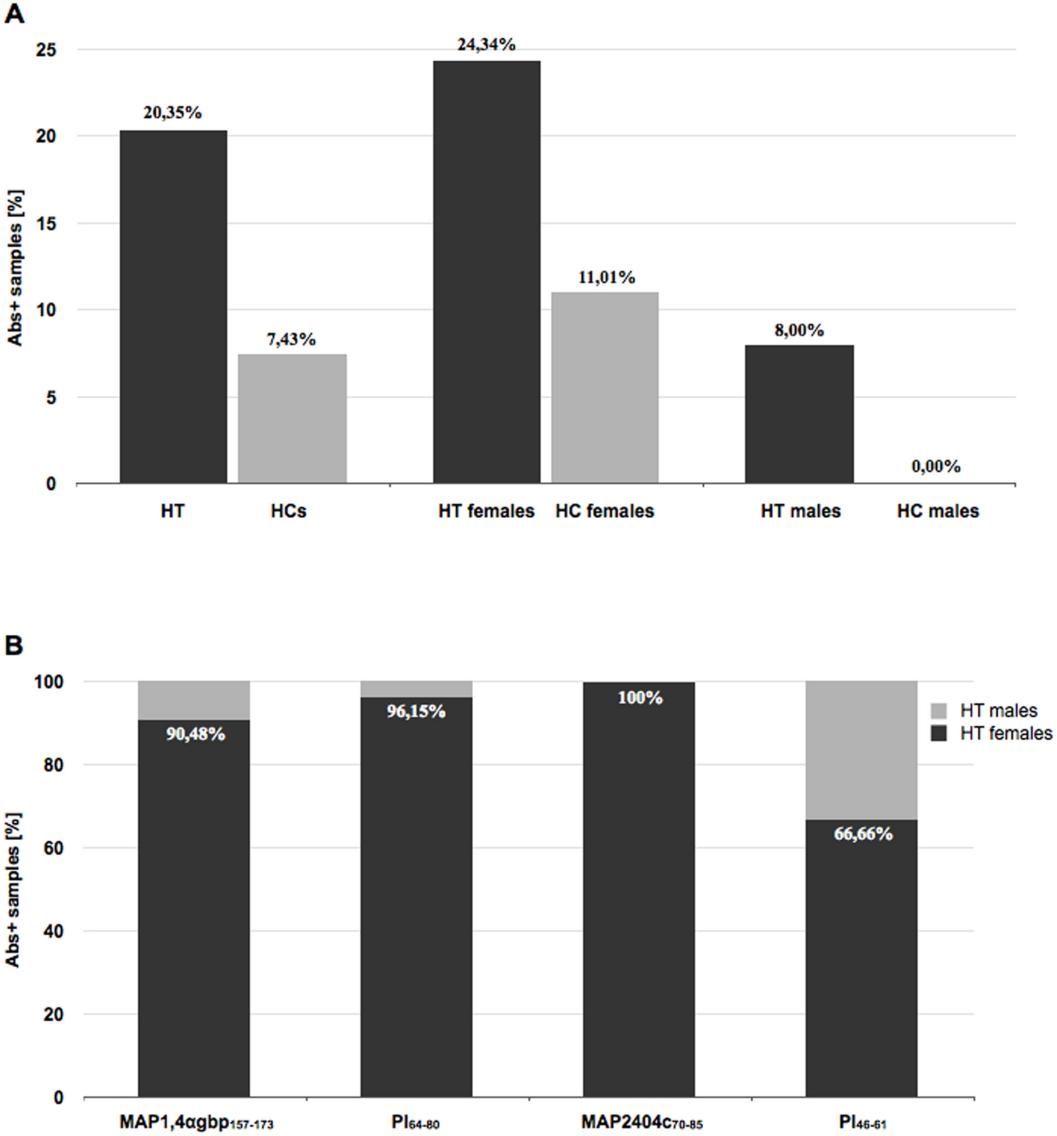
Prevalence of serum Ab positivity to MAP1,4αgbp_157-173_/PI_64-80_ and MAP2404c_70-85_/PI_46-61_ homologus epitopes in Sardinian HT patients and age/sex-matched HCs.

**Fig 2:**
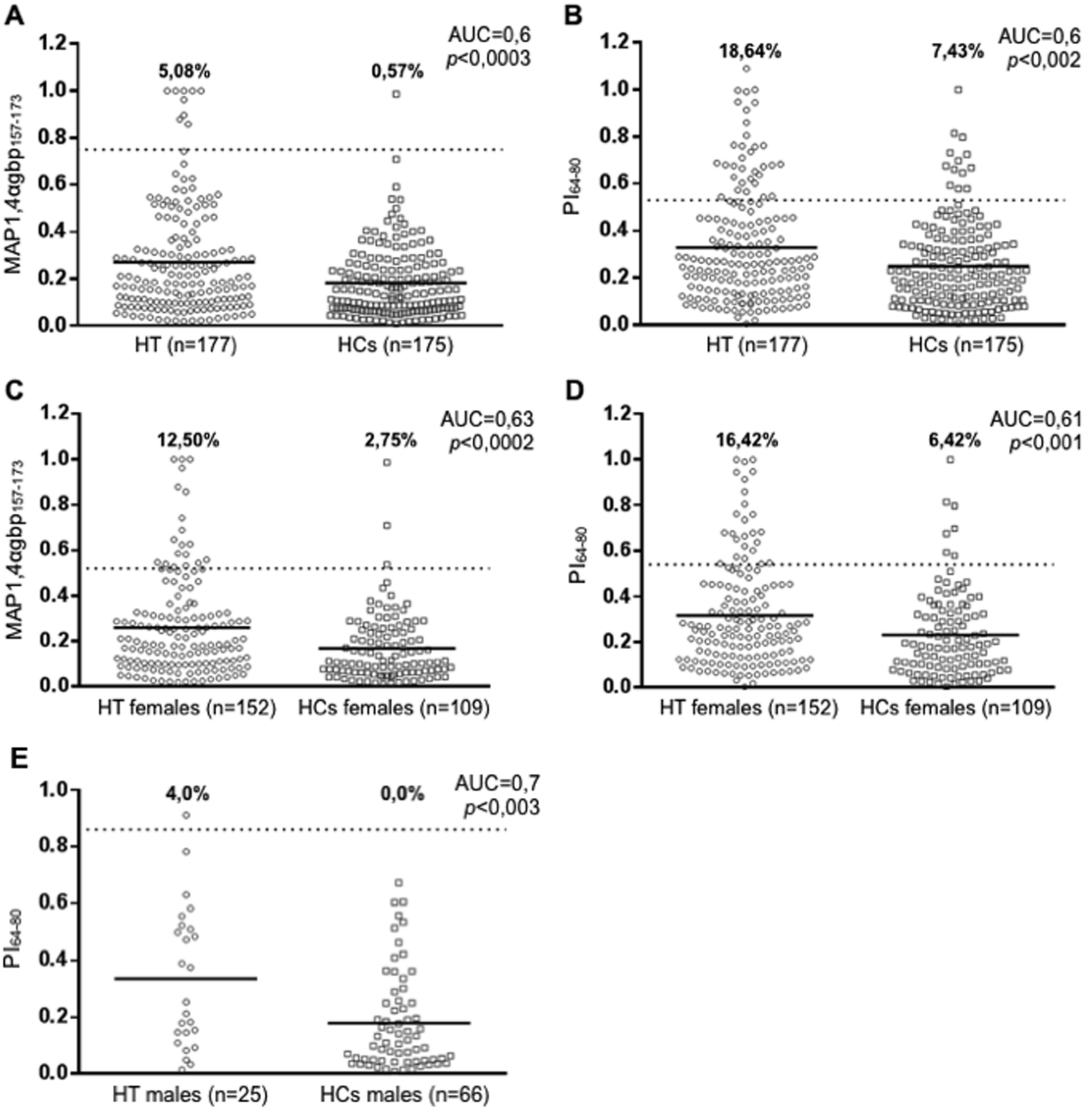
Sex-related prevalence of Abs against MAP1,4αgbp_157-173_/PI_64-80_ and PI_46-61_ in Sardinian HT patients and HCs. Plasma was tested for immunoreactivity against plate-coated homologous peptides MAP1,4αgbp_157-173_/PI_64-80_ and MAP2404c_70-85_/PI_46-61_. Statistical analyses were performed for all peptides separately in the context of entire study population (A, B), female (C, D) and male (E) HT subjects and HCs. The dotted lines indicate thresholds of positivity relative to each assay calculated by ROC analysis. The percentage of Abs-positive patients is reported on top of each distribution; horizontal bars correspond to means. AUC and *p* values (CI 95%) are indicated in the top right corner. Only statistically significant data are reported.

Among 177 HT patients, 20,34% (n = 33) showed positivity to at least one of the four assessed peptides (Fig 1A). 48,5% of positive subjects had Abs against at least two peptides, 87,5% of which were positive to any of the homologous peptide pairs. One HT patient was reactive to all analysed peptides. When single-peptide analyses of Ab reactivity were performed, 9 HT patients resulted positive to MAP1,4αgbp_157-173_ (5,08%) compared to only 0,57% of HCs (AUC = 0,6; *p*<0,0003) (Fig 2A). The homologous PI_64-80_ peptide was recognized by 33 patients (18,64%) and 7,43% of HCs (AUC = 0,6; *p*<0,002) (Fig 2B).

Serum Ab reactivity to MAP2404c_70-85_ and its homolog PI_46-61_ was observed in 6 (3,39%) and 7 (3,95%) HT patients, respectively, with no Ab against both peptides detected in HCs, however the differences were not significant.

Considering positive cases, a higher immunoreactivity to both homologous peptide pairs was found among women, accounting for 90,48% for MAP1,4αgbp_157-173_, 96,15% for PI_64-80_, 100% for MAP2404c_70-85_ and 66,66% for PI_46-61_ (Fig 1B). Upon sex-based analysis of the study population, prevalence of positivity to MAP1,4αgbp_157-173_/PI_64-80_ and MAP2404c_70-85_ was markedly higher among females, while responses to PI_46-61_ were slightly higher in men.

In women (n = 152), Abs against MAP1,4αgbp_157-173_ were detected in 12,50% of HT subjects and in 2,75% of HCs (AUC = 0,63; *p*<0,0002) (Fig 2C). Positivity to its human homolog PI_64-80_ was observed in 16,42% of patients, compared to 6,42% of HCs (AUC = 0,61; *p*<0,001) (Fig 2D). Even though anti-MAP2404c_70-85_ Abs were identified in 9,21% of HT patients and in 4,59% of HCs, while 1,32% of female patients and none of sex-matched HCs resulted positive to the homologous PI_46-61_, statistical significance was not attained for this peptide pair.

Males (n = 25) displayed higher but not significant levels of Abs against the homologous MAP1,4αgbp_157-173_ and PI_64-80_ giving 8% and 4% of positive patients, respectively, compared to HCs among which no Abs were detected. Serum reactivity was not observed for MAP2404c_70-85_ either in HT patients or HCs, whereas 4% of male patients and none of the HC subjects resulted positive to the homologous PI_46-61_ peptide (AUC = 0,7; *p*<0,003) (Fig 2E).

Six patients had diseases of non-autoimmune origin complicating HT: one patient positive to the first peptide pair had hepatitis C; one out of three HT women with hypertension responded to MAP1,4αgbp_157-173_ and showed anti-PI_64-80_ reactivity slightly below the fixed positivity threshold; two patients with type 2 diabetes did not result positive to any of the anti-MAP/PI Abs. Three HT patients had concomitant autoimmune diseases: one with allergic asthma did not show significant levels of Abs against any of the studied peptides; one male patient with coeliac disease (16 years) responded to MAP1,4αgbp_157-173_/PI_64-80_ peptide pair and had Abs values close to the cut-off for MAP2404c_70-85_; one HT female with psoriasis (33 years) was positive to Abs against PI_46-61_.

### Prevalence of Abs against MAP1,4αgbp and MAP2404c/PI peptides in HT and sex-matched HC age goups

Analyses of trends in age prevalence relative to Abs directed against any of the studied peptides revealed differences in positivity among HT patients’ age groups: <20 years (22,22%), 20–30 years (15,38%), 31–40 years (45,83%), 41–50 years (10,42%), 51–60 years (23,81%) and >60 years (13,33%). Detailed data are presented in Table 1.

**Table 1:** Age-related trends in prevalence of anti-MAP/PI Abs+ Sardinian HT patients. Values are reported as percentage of positive cases. PR: prevalence ratio calculated in relation to HCs; PR is not indicated when Abs+ cases were not found among HCs. Absence of Abs+ cases is marked by hyphens.

HT subjects	Peptides	Age groups (years)									
		<20	20–30	PR	31–40	PR	41–50	PR	51–60	PR	>60
Total study population	MAP/PI 4 peptides	22,22	15,38	1,12	45,83	3,01	10,42	2,5	23,81		13,33
	MAP1,4αgbp_157-173_	16,67	-		4,16		2,08		7,14		3,33
	PI_64-80_	22,22	15,38	1,12	48,67	3,2	10,42	2,5	21,43		10
	MAP2404c_70-85_	-	7,69		-		2,08		2,38		6,67
	PI_46-61_	11,11	-		4,16		2,08		4,76		3,33
Females	MAP/PI 4 peptides	18,18	16,67	0,83	33,33	2,08	17,07	2,56	42,11	8,85	11,54
	MAP1,4αgbp_157-173_	18,18	8,33	2,08	14,28	3,57	7,31		23,68	4,97	3,85
	PI_64-80_	18,18	16,67	1,04	33,33	2,78	9,76		21,05		7,69
	MAP2404c_70-85_	-	8,33	1,04	4,76	1,19	9,76	1,46	15,79		7,69
	PI_46-61_	9,09	-		-		-		-		3,85
Males	MAP/PI 4 peptides	14,28	-		33,33		-		-		-
	MAP1,4αgbp_157-173_	14,28	-		33,33		-		-		-
	PI_64-80_	14,28	-		-		-		-		-
	MAP2404c_70-85_	-	-		-		-		-		-
	PI_46-61_	14,28	-		-		-		-		-

Anti-PI_64-80_ Abs were prevalent in each age group showing the highest positivity (48,67%) in patients 31 to 40 years comparing to 15,22% among HCs (AUC = 0,65; *p*<0,03), followed by 21,43% of immunoreactivity among 51–60 year-old HT subjects (AUC = 0,74; *p*<0,0002) and no responses registered for HCs. The homologous MAP1,4αgbp_157-173_ peptide was recognized by 4,16% (AUC = 0,64; *p*<0,05) and 7,14% (AUC = 0,73; *p*<0,0004) of patients from the respective age groups, while positive cases were not detected among HCs.

For the same age groups, 4,16% (AUC = 0,64; *p*<0,04) and 4,76% (AUC = 0,63; *p*<0,04) of HT patients displayed positivity to PI_46-61_. Abs directed against its homolog MAP2404c_70-85_ were not observed among 31–40 year-old patients (AUC = 0,67, *p*<0,01), but 2,38% of HT subjects 51 to 60 years resulted reactive (AUC = 0,69; *p*<0,004). All HCs were negative for this peptide pair.

In female patients, the highest prevalence of positivity to the studied peptides occurred for 51–60 years group and accounted for 42,11%. In particular, a significant immunoreactivity to MAP1,4αgbp_157-173_/PI_64-80_ homologs was observed in 23,68% (AUC = 0,68; *p*<0,016) and 21,05% (AUC = 0,69; *p*<0,008) of HT women, respectively, compared to 4,76% of positivity to MAP1,4αgbp_157-173_ and no anti-PI_64-80_ Abs detected among female HCs. Highly significant results of Ab prevalence for the same peptide pair were obtained for 41–50 years group with 7,31% of female patients positive to MAP1,4αgbp_157-173_ (AUC = 0,68; *p*<0,009) and 9,76% to the homologous PI_64-80_ (AUC = 0,7; *p*<0,003), whereas responses to these peptides were not detected in HC women.

Seropositivity among HT males was highest (33,33%) between 31 to 40 years of age due to the presence of anti-MAP1,4αgbp_157-173_ Abs (AUC = 0,9; *p*<0,02). Positive cases were not detected among HC men for any of the analysed peptides in all age groups.

### Prevalence of anti-MAP/PI Abs in correlation with anti-TPO and anti-Tg Abs, levels of TSH, FT3 and FT4 in HT subjects

Even though 80% of HT patients reactive to MAP/PI epitopes had anti-TPO Abs levels above positivity threshold, prevalence of MAP/PI peptides among anti-TPO negative subjects accounted for 60% compared to 27% among anti-TPO positive ones. A higher prevalence (36%) was observed for anti-TPO 100–1000 UI/mL range and decreased to 15% for >1000 UI/mL values.

All HT patients positive to MAP/PI peptides had anti-TG Abs above the 10 UI/mL cut-off with 32% prevalence and a homogeneous distribution up to 5000 UI/mL.

67% of MAP/PI immunoreactive HT subjects had TSH levels below or above the normal range values (0,46–4,68 μU/ml) with an overall prevalence of 29%. Prevalence relative to <0,46 μU/ml TSH accounted for 20% and for 32% when values exceeded 4,68 μU/ml. Patients with normal TSH values showed 19% prevalence of anti-MAP/PI Abs positivity.

Only 7% of HT patients responding to MAP/PI peptides had FT3 levels exceeding the 5,26 pg/ml threshold, however the prevalence reached 33% compared to 21% for subjects with normal FT3 values.

FT4 levels fell outside the normal range of 0,77–2,19 ng/dl in 14% of MAP/PI-positive HT subjects, with 33% and 50% prevalence for values below and above the reference thresholds, respectively. Prevalence of MAP/PI reactivity among patients with normal FT4 values equaled 21%.

Anti-TPO Abs concentrations moderately correlated with both MAP peptides in MAP/PI positive HT subjects, however statistical significance was not attained. Upon sex-related analyses, males presented a high correlation with PI_46-61_ (0,89; *p*<0,042); the remaining peptides correlated moderately or even highly for MAP1,4αgbp_157-173_, yet without significance. Moderate but not significant correlation with MAP epitopes was found among females.

Although age-related analysis did not produce significant results, a moderately high correlation of anti-TPO Abs with MAP1,4αgbp_157-173_/PI_64-80_ homologous peptides was detected in the 31–40 year-old group. No correlation with anti-TG Abs concentrations and levels of TSH, FT3 and FT4 was found for any of the analyzed peptide pairs.

## Discussion

We investigated the presence of Abs directed against proinsulin and homologous MAP-derived peptides in Sardinian HT patients. The present study demonstrated a significant association of anti-PI and anti-MAP Abs in the analysed subjects, suggesting a possible role of MAP in T1D pathogenesis when occurring as an associated disorder of HT.

HT is the most frequent organ-specific autoimmune disorder complicating T1D by involvement of common immunological processes shared by different autoimmune diseases [2]. In Sardinian population, AITD is a prevailing autoimmune disease, while T1D is well known to have the second highest prevalence worldwide [21]. Our group previously described a markedly significant association of MAP with T1D in Italian pediatric patients, analyzed through detection of seroreactivity against identical MAP/PI homologous peptides as those used in the present study [18]. Positivity to both peptides in HT subjects is detected in most cases within the same person providing another indication that homologous MAP and PI regions may be cross-recognized by human Abs. Even though comorbidity and familial aggregation of autoimmune diseases envision common genetic determinants, substantial co-existence within siblings compared to incidence between successive generations highlights environmental impact [22]. In the present study, 54,38% of patients with complete clinical data had HT history in family. This ratio reached 70% among HT subjects positive to any of the analyzed peptides, suggesting a possible transmission of MAP, MAP-derived peptides or anti-MAP Abs within family.

While women are 5–10 times more affected by HT [23], there is no T1D sex-prevalence in childhood, although morbidity among men of European origin is more common in early adulthood [24]. Our results are partially consistent with these trends, accounting for almost 86% of women in the HT study population and showing a higher prevalence of anti-MAP and anti-PI Abs among adult females. 5% prevalence of GADA in HT non-diabetic patients was reported not to impaire insulin action or secretion [25]. Prevalence to three out of four peptides analyzed in our study was up to 5%, however positivity to PI_64-80_ exceeding 18% of HT patients could increase the risk for adult-onset diabetes. Moriguchi et al. documented a high preavlence of seropositivity to glutamic acid decarboxylase (GAD) in Japanese patients with AITD when compared to HCs and patients with thyroiditis of non-autoimmune origin, however the major contribution was attributed to Grave’s disease (GD), whereas HT patients presented only slight but not significant GADA prevalence [26]. The authors suggested that AITD patients with high GADA titers and positivity to multiple islet autoantibodies are at risk for the development of T1D. Similarly, Nuovo et al. reported no significant positivity for IAA and anti-insulin receptor Abs (AIRA) in HT patients but high levels of both aAbs were typical for GD patients [27]. In contrast, anti-IAA were detected by other authors [28–30], even though a further study describes high responses to GADA in non-diabetic HT patients with negative association to IAA [31].

Interestingly, age-related analyses revealed a markedly higher prevalence of anti-MAP/PI Abs in HT patients 31 to 40 years old indicating a possible association with latent autoimmune diabetes in adults (LADA), even though IAA tend to be undetectable in late-onset T1D. This fact points at a putative involvement of MAP as an environmental agent contributing to the development of autoimmunity and permits to hypothesize a cross-reaction between the homologous peptides. In HT females, this trend was switched to the period of 51–60 years old, although a significant prevalence was already visible in 41–50 age group. This trend is typical for age-related incidence of type 2 diabetes, however we previously demonstrated no association of MAP with T2D [32]; similarly, two HT patients with concomitant T2D included in the present study resulted negative to both MAP/PI peptide pairs. High prevalence of anti-MAP Abs in older groups of Irish cattle affected by Johne’s disease [33] may indicate a similar time-depending natural scenario of immune responses in MAP-positive subjects. Finally, the onset of autoimmune diseases in males is supposed to occur earlier compared to females and is characterized by acute inflammation and the appearance of aAbs [34]. In the study population, mean anti-MAP/PI Abs titers among positive patients were higher in men.

Regardless a lower prevalence of anti-MAP/PI Abs in children and youth (<20 years), two HT patients had very high Abs values for three peptides. The same Abs are demonstrated to appear before the classical anti-islet aAbs in Sardinian children at onset of T1D (unpublished data), therefore, may suggest a developing β-cell autoimmunity.

Presence of anti-MAP/PI Abs in two out of three HT patients with concomitant autoimmune diseases may point at prediction of multiple autoimmune syndrome (MAS), however such an association should be investigated in a wider group. Since dermatological conditions have an important place in MAS, it would be of particular relevance for patients with psoriasis, the most frequent autoimmune disease affecting Sardinian men.

The analysis of clinical data showed reactivity to MAP/PI epitopes among HT patients to be associated with the presence of anti-TG Abs and high TSH levels. Although FT3 and FT4 presented a high prevalence outside the reference range, only a few cases fell within this group. Anti-TPO Abs were detected in 80% of MAP/PI-positive patients and revealed a moderate correlation with concentrations of anti-MAP Abs; a high prevalence of Abs against the homologous peptides among anti-TPO-negative HT subjects requires evaluation in a wider number of patients. For this thyroid biomarker, correlation with proinsulin became even stronger when analyzed separately in males. Interestingly, low anti-TPO Abs concentrations corresponded to higher values obtained for Abs directed against MAP peptides; this combination may indicate a possible protective link against raising anti-TPO Abs titers, especially between 31 and 40 years, but could be confirmed following to further investigation of Abs dynamics in the analyzed patients. Conversely, Abs against proinsulin correlated positively with increasing anti-TPO Abs concentrations in men suggesting a probable risk for development of autoimmune diabetes in adolescents and young adults.

In conclusion, we here provided the first evidence of significantly high reactivity to proinsulin and MAP-derived homologous peptides in the context of HT, with distinct prevalence in 51–60 year-old women and in 31–40 year-old men. A prospective follow-up of positive patients and analyses of classical anti-islet aAbs levels will permit to evaluate the association of immune responses directed against the homologous peptides with progression to overt T1D in HT subjects. Likewise, diet composition, in particular consumption of dairy products and iodine rich food, as well as genetic polymorphisms need to be assessed in relation to anti-MAP/PI positivity. Moreover, detection of anti-MAP/PI Abs in T1D adult patients would provide an additional view on age/sex-related prevalence.

## References

[1] Kawasaki E. Type 1 Diabetes and Autoimmunity. Clin Pediatr Endocrinol. 2014 10;23(4):99–105. 10.1297/cpe.23.99 Epub 2014 Nov 6. 25374439 PMC4219937

[2] Mantovani RM, Mantovani LM, Dias VM. Thyroid autoimmunity in children and adolescents with type 1 diabetes mellitus: prevalence and risk factors. J Pediatr Endocrinol Metab. 2007 6;20(6):669–75. 17663291 10.1515/jpem.2007.20.6.669

[3] Korodonouri O, Klinghammer A, Lang EB, Grüters-Kieslich A, Grabert M, Holl RW. Thyroid autoimmunity in children and adolescents with type 1 diabetes: a multicenter survey. Diabetes Care. 2002 8;25(8):1346–50. 12145233 10.2337/diacare.25.8.1346

[4] Bright GM, Blizzard RM, Kaiser DL, Clarke WL. Organ-specific autoantibodies in children with common endocrine diseases. J Pediatr. 1982 1;100(1):8–14. 7035635 10.1016/s0022-3476(82)80227-8

[5] Severinski S, Banac S, Severinski NS, Ahel V, Cvijović K. Epidemiology and clinical characteristics of thyroid dysfunction in children and adolescents with type 1 diabetes. Coll Antropol. 2009 3;33(1):273–9. 19408637

[6] Korodonouri O, Hartmann R, Deiss D, Wilms M, Grüters-Kieslich A. Natural course of autoimmune thyroiditis in type 1 diabetes: association with gender, age, diabetes duration, and puberty. Arch Dis Child. 2005 4;90(4):411–4. 15781936 10.1136/adc.2004.056424 PMC1720371

[7] Cooper GS, Stroehla BC. The epidemiology of autoimmune diseases. Autoimmun Rev. 2003 5;2(3):119–25. 12848952 10.1016/s1568-9972(03)00006-5

[8] Eaton WW, Rose NR, Kalaydjian A, Pedersen MG, Mortensen PB. Epidemiology of autoimmune diseases in Denmark. J Autoimmun. 2007 8;29(1):1–9. Epub 2007 Jun 19. 17582741 10.1016/j.jaut.2007.05.002 PMC2717015

[9] Somers EC, Thomas SL, Smeeth L, Hall AJ. Autoimmune diseases co-occurring within individuals and within families: a systematic review. Epidemiology. 2006 3;17(2):202–17. 10.1097/01.ede.0000193605.93416.df 16477262

[10] Henkin RI. Trace metals in endocrinology. Med Clin North Am. 1976 7;60(4):779–97. 775219 10.1016/s0025-7125(16)31861-2

[11] Chimienti F, Devergnas S, Pattou F, Schuit F, Garcia-Cuenca R, Vandewalle B, et al In vivo expression and functional characterization of the zinc transporter ZnT8 in glucose-induced insulin secretion. J Cell Sci. 2006 10 15;119(Pt 20):4199–206. Epub 2006 Sep 19. 16984975 10.1242/jcs.03164

[12] Wenzlau JM, Juhl K, Yu L, Moua O, Sarkar SA, Gottlieb P, et al The cation efflux transporter ZnT8 (Slc30A8) is a major autoantigen in human type 1 diabetes. Proc Natl Acad Sci USA. 2007 10 23;104(43):17040–5. Epub 2007 Oct 17. 17942684 10.1073/pnas.0705894104 PMC2040407

[13] Rogowicz-Frontczak A, Zozuliłska-Ziołkiewicz D, Litwinowicz M, Niedźwiecki P, Wyka K, Wierusz-Wysocka B. Are zinc transporter type 8 antibodies a marker of autoimmune thyroiditis in non-obese adults with new-onset diabetes? Eur J Endocrinol. 2014 3 14;170(4):651–8. 10.1530/EJE-13-0901 Print 2014 Apr. 24480135

[14] Masala S, Cossu D, Palermo M, Sechi LA. Recognition of Zinc Transporter 8 and MAP3865c Homologous Epitopes by Hashimoto’s Thyroiditis Subjects from Sardinia: A Common Target with Type 1 Diabetes? PLoS One. 2014 5 15;9(5):e97621 10.1371/journal.pone.0097621 eCollection 2014. 24830306 PMC4022723

[15] Masala S, Paccagnini D, Cossu D, Brezar V, Pacifico A, Ahmed N, et al Antibodies Recognizing Mycobacterium avium paratuberculosis Epitopes Cross-React with the Beta-Cell Antigen ZnT8 in Sardinian Type 1 Diabetic Patients. PLoS One. 2011;6(10):e26931 10.1371/journal.pone.0026931 Epub 2011 Oct 27. 22046415 PMC3203182

[16] Masala S, Cossu D, Piccinini S, Rapini N, Massimi A, Porzio O, et al Recognition of zinc transporter 8 and MAP3865c homologous epitopes by new-onset type 1 diabetes children from continental Italy. Diabetol. 2014 8;51(4):577–85. 10.1007/s00592-014-0558-2 Epub 2014 Feb 5. 24496951

[17] Masala S, Zedda MA, Cossu D, Ripoli C, Palermo M, Sechi LA. Zinc Transporter 8 and MAP3865c Homologous Epitopes are Recognized at T1D Onset in Sardinian Children. PLoS One. 2013 5 17;8(5):e63371 10.1371/journal.pone.0063371 Print 2013. 23696819 PMC3656963

[18] Masala S, Cossu D, Piccinini S, Rapini N, Mameli G, Manca Bitti ML, et al Proinsulin and MAP3865c homologous epitopes are a target of antibody response in new-onset type 1 diabetes children from continental Italy. Pediatr Diabetes. 2015 2 27 10.1111/pedi.12269 [Epub ahead of print] 25720593

[19] Culina S, Brezar V, Mallone R. Insulin and type 1 diabetes: immune connections. Eur J Endocrinol. 2013 1 17;168(2):R19–31. 10.1530/EJE-12-0693 Print 2013 Feb. 23065992

[20] Ziegler AG, Bonifacio E, the BABYDIAB-BABYDIET Study Group. Age-related islet autoantibody incidence in offspring of patients with type 1 diabetes. Diabetologia. 2012 7;55(7):1937–43. 10.1007/s00125-012-2472-x 22289814

[21] Sardu C, Cocco E, Mereu A, Massa R, Cuccu A, Marrosu MG, et al Population based study of 12 autoimmune diseases in Sardinia, Italy: prevalence and comorbidity. PLoS One. 2012;7(3):e32487 10.1371/journal.pone.0032487 Epub 2012 Mar 2. 22396771 PMC3292563

[22] Cooper GS, Bynum MLK, Somers EC. Recent Insights in the Epidemiology of Autoimmune Diseases: Improved Prevalence Estimates and Understanding of Clustering of Diseases. J Autoimmun. 2009 Nov-Dec;33(3–4):197–207. 10.1016/j.jaut.2009.09.008 Epub 2009 Oct 9. 19819109 PMC2783422

[23] Vanderpump MP, Tunbridge WM, French JM, Appleton D, Bates D, Clark F, et al The incidence of thyroid disorders in the community: a twenty-year follow-up of the Whickham Survey. Clin Endocrinol (Oxf). 1995 7;43(1):55–68. 7641412 10.1111/j.1365-2265.1995.tb01894.x

[24] Wändell PE, Carlsson AC. Time trends and gender differences in incidence and prevalence of type 1 diabetes in Sweden. Curr Diabetes Rev. 2013 7;9(4):342–9. 23721159 10.2174/15733998113099990064

[25] Aksoy DY, Yürekli BP, Yildiz BO, Gedik O. Prevalence of glutamic acid decarboxylase antibody positivity and its association with insulin secretion and sensitivity in autoimmune thyroid disease: A pilot study. Exp Clin Endocrinol Diabetes. 2006 9;114(8):412–6. 17039421 10.1055/s-2006-924153

[26] Moriguchi M, Noso S, Kawabata Y, Yamauchi T, Harada T, Komaki K, et al Clinical and genetic characteristics of patients with autoimmune thyroid disease with anti-islet autoimmunity. Metabolism. 2011 6;60(6):761–6. 10.1016/j.metabol.2010.07.025 Epub 2010 Sep 9. 20825955

[27] Nuovo JA, Baker JR Jr, Wartofsky L, Lukes YG, Burman KD. Autoantibodies to insulin are present in sera of patients with autoimmune thyroid disease. Diabetes. 1988 3;37(3):317–20. 3286332 10.2337/diab.37.3.317

[28] Di Mario U, Perfetti R, Anastasi E, Contreas G, Crisà L, Tiberti C, et al Autoantibodies to insulin do appear in non-diabetic patients with autoimmune disorders: comparison with anti-immunoglobulin antibodies and other autoimmune phenomena. Acta Endocrinol (Copenh). 1990 3;122(3):303–8. 2183533 10.1530/acta.0.1220303

[29] Hegewald MJ, Schoenfeld SL, McCulloch DK, Greenbaum CJ, Klaff LJ, Palmer JP. Increased specificity and sensitivity of insulin antibody measurements in autoimmune thyroid disease and type I diabetes. J Immunol Methods. 1992 9 18;154(1):61–8. 1401944 10.1016/0022-1759(92)90213-d

[30] Vardi P, Modan-Mozes D, Ish-Shalom S, Soloveitzik L, Barzilai D, Modan M. Low titer, competitive insulin autoantibodies are spontaneously produced in autoimmune diseases of the thyroid. Diabetes Res Clin Pract. 1993 Aug-Sep;21(2–3):161–6. 8269817 10.1016/0168-8227(93)90064-c

[31] Kawasaki E, Abiru N, Yano M, Uotani S, Matsumoko K, Matsuo H, et al Autoantibodies to Glutamic Acid Decarboxylase in Patients with Autoimmune Thyroid Disease: Relation to Competitive Insulin Autoantibodies. J Autoimmun. 1995 10;8(5):633–43. 8579720 10.1006/jaut.1995.0047

[32] Rosu V, Niyaz A, Paccagnini D, Pacifico A, Zanetti S, Sechi LA. Mycobacterium avium subspecies paratuberculosis is not associated with Type-2 Diabetes Mellitus. Ann Clin Microbiol Antimicrob. 2008 4 22;7:9 10.1186/1476-0711-7-9 18430197 PMC2365959

[33] Good M, Clegg T, Sheridan H, Yearsely D, O’Brien T, Egan J, et al Prevalence and distribution of paratuberculosis (Johne's disease) in cattle herds in Ireland. Ir Vet J. 2009 9 1;62(9):597–606. 10.1186/2046-0481-62-9-597 21851740 PMC3113810

[34] Fairweather D, Frisancho-Kiss S, Rose NR. Sex Differences in Autoimmune Disease from a Pathological Perspective. Am J Pathol. 2008 9; 173(3): 600–609. 10.2353/ajpath.2008.071008 18688037 PMC2527069

